# Follow up after Primary Treatment of Soft Tissue Sarcoma: A Survey of Current Practice in the United Kingdom

**DOI:** 10.1155/2007/34128

**Published:** 2007-12-26

**Authors:** C. H. Gerrand, L. J. Billingham, P. J. Woll, R. J. Grimer

**Affiliations:** ^1^ North of England Bone and Soft Tissue Tumour Service, Freeman Hospital, Newcastle Upon Tyne Hospitals, Newcastle NE7 7DN, UK; ^2^ Cancer Research UK Clinical Trials Unit, University of Birmingham, Division of Cancer studies, Edgbaston, Birmingham B15 2TT, UK; ^3^ Section of Oncology and Pathology, Division of Genomic Medicine, University of Sheffield, Sheffield S10 2SJ, UK; ^4^ Orthopaedic Oncology Service, The Royal Orthopaedic Hospital, Northfield, Birmingham B31 2AP, UK

## Abstract

Despite the clinical and financial implications, there is little evidence about how patients who have been treated for soft tissue sarcoma should be followed up. The purpose of this study was to determine current practice in the United Kingdom. 192 clinicians treating patients with soft tissue sarcoma were surveyed with a postal questionnaire enquiring about frequency and method of follow up and how patients would be followed up in each of 3 clinical scenarios: a patient with a trunk or extremity tumour at low risk of relapse; a patient with a trunk or extremity tumour at high risk of relapse; and a patient with a retroperitoneal or abdominal tumour. 155 (81%) clinicians responded. Clinic visits and X-rays were the most frequently used methods of follow up. Chest CT scans, local site imaging, and blood tests were used infrequently. The intensity and methods of follow up varied with each of the clinical scenarios. There was a seven-to-twenty fold variation in cost between the least and the most expensive regimes. Respondents were generally supportive of the development of the clinical trial in this area.

## 1. INTRODUCTION

Soft tissue sarcomas are a heterogenous group of rare tumours of mesenchymal or neuroectodermal origin. They can occur in any anatomical location, with around 55% in the trunk or extremities, 35% in the retroperitoneum or viscera, and 10% in the head and neck (Pollock et al. [1]). Surgical excision with or without adjuvant radiotherapy is the treatment of choice for localised disease (Yang et al. [2]). The role of adjuvant chemotherapy remains unproven (Tierney et al. [3]). There is a significant rate of relapse following primary treatment, with 10-year local recurrence rate of 10–20% and 10-year disease-specific survival rate of 50–60% in published series (Eilber et al. [4]; Kattan et al. [5]; Trovik [6]). Retroperitoneal tumours are associated with poorer disease-specific survival than tumours in the extremity (Kattan et al. [5]). Prognostic factors for tumour relapse are well documented but complex, as different factors contribute to the risk of local and distant recurrence. Despite evidence that some patients who relapse can be salvaged, and that different follow-up practices may affect patient outcome and have significant financial implications, few studies have looked at how patients with soft tissue sarcoma should be followed up.

A survey of members of the Society of Surgical Oncology (Ill, USA) demonstrated significant variation in follow-up protocols after treatment of soft tissue sarcoma (Beitier et al. [7]). The estimated costs of follow up were shown to vary between protocols by a factor of 43 (Goel et al. [8]). However, little is known about current practice in the United Kingdom, where most cancer care is delivered within the National Health Service. A survey of UK clinicians was undertaken to determine how patients with soft tissue sarcoma are followed up in the United Kingdom. This paper reports the results from this survey, which will inform the development of guidelines about follow up and further clinical studies in this area.

## 2. MATERIALS AND METHODS

A cross-sectional survey was carried out in 2004 on behalf of the National Cancer Research Institute (NCRI) Sarcoma Clinical Studies Group. The target population comprised clinicians treating patients with soft tissue sarcoma in the United Kingdom. There is no definitive list of these clinicians available, and therefore a list was compiled from records held by the British Sarcoma Group, the Sarcoma-UK support group, and a web-based directory of hospital specialists (www.specialistinfo.com). The authors reviewed the final list for errors or omissions.

The survey instrument was a self-completed questionnaire administered by post. It was based on, but not identical to the survey performed by Johnson et al., (Beitier et al. [7]; Johnson et al. [9]; Sakata et al. [10, 11]). The questionnaire comprised 31 items in three sections. The first section contained items asking about the clinical practice and specialty interests of respondents. The second section asked about the follow-up practices of respondents and requested specific information about how patients in three clinical scenarios would be followed up. These scenarios were as follows: a patient with a trunk or extremity tumour at low risk of relapse; a patient with a trunk or extremity tumour at high risk of relapse; and a patient with a retroperitoneal or intraabdominal tumour. Questions included the overall length of follow up, the number of outpatient visits, chest x-rays, chest CT scans, and local site imaging investigations (USS, CT, or MRI) performed in each of the first 5 years of follow up and in each year thereafter. In the third section, respondents were asked about their motivation for following patients up and about opinions of a proposed study of follow-up protocols.

Before distribution, the questionnaire was piloted informally amongst a small group of clinicians. To maximise the response rate, the initial mailing included a single questionnaire with covering letter and a stamped addressed envelope. Reminder letters were sent at two, four, and six weeks, with a replacement questionnaire at four weeks. Some nonresponders were also contacted by email or telephone.

### 2.1. Cost analysis

For each of the three different clinical scenarios, the responses given by each clinician for number of outpatient visits, chest x-rays, chest CT scans, and local site imaging investigations during follow up were multiplied by unit costs for these resource usage items to give a total cost for follow up. It was assumed that patients followed to “adulthood” or “lifelong” were followed for a total of 10 years, and the patient survived to the end of the maximum follow up period and that local site imaging, where used, was an MRI scan. The cost of blood tests was not included, and the costs of treatment of a detected relapse were not considered. Unit costs were taken from the Department of Health National Reference Cost Index (Health [12]) (London, UK) and were costed at £62 for an outpatient visit, £87 for a chest x-ray, £179 for a chest CT scan, and £285 for an MRI.

## 3. RESULTS

### 3.1. Response rate to survey

A list of 192 names from 45 different centres was compiled. Questionnaires were returned by 155 of those originally surveyed, giving an overall response rate of 81%. Of these 155, twenty-one respondents were not involved in the treatment of soft tissue sarcomas, three had retired, three could not be traced, and seven were returned blank leaving 121 questionnaires from 43 different centres available for analysis. These are referred to as the survey respondents.

### 3.2. Characteristics and practice of survey respondents

Of the 121 survey respondents, 32 were clinical oncologists, 30 orthopaedic surgeons, 26 paediatric oncologists, 16 medical oncologists, 11 general surgeons, and 6 were plastic surgeons. There were 119 consultants, one staff grade, and one registrar. Within the survey respondents, 74 (61%) declared membership of at least one specialist society related to the treatment of sarcomas.

Management of patients with soft tissue sarcoma formed more than 50% of clinical workload in 13 (11%) survey respondents, between 11% and 50% of clinical workload in 53 (44%) and less than 10% of clinical workload in 54 (45%) (1 not specified). The majority of the survey responders (110; 91%) were responsible for the long-term follow up of patients with soft tissue sarcoma and most (110; 91%) had access to a multidisciplinary team dealing with sarcomas.

### 3.3. Perceived risk factors for relapse

Seventy-five of 100 responders (75%) stated their follow-up protocol for adult patients with soft tissue sarcoma depended upon the perceived risk of local or systemic relapse (21 did not answer this question due to lack of involvement either in long-term follow up or in adult patients). Responders were asked which of a choice of factors (histological grade, unplanned excision before referral, tumour size, surgical margins, age over 50 years, deep location, histological type) they considered significantly increased the risk of local recurrence and metastatic disease in patients with soft tissue sarcoma. Of the 107 respondents who answered this question (14 nonresponders due to lack of involvement either in long-term follow up or in adult patients), surgical margin status (96%), histological grade (89%), and tumour size (87%) were the three most commonly chosen risk factors for local recurrence, whereas histological grade (94%), tumour size (83%), and histological type (77%) were the three most commonly chosen risk factors for metastatic disease. In both cases, age over 50 years was lowest in popularity as a risk factor (14% for local recurrence and 13% for metastatic disease). Other risk factors mentioned included site of disease, previous recurrence at site, adjuvant therapy, and genetics.

### 3.4. Actual follow-up practice for different clinical scenarios

The second part of the questionnaire related to three different clinical scenarios. Because of variation in the patients treated by survey respondents, 88 were able to answer questions about the follow up of patients with trunk or extremity tumours at low risk of relapse, 99 about patients with high-risk trunk or extremity tumours, and 63 about patients with retroperitoneal or intra-abdominal tumours. Length of follow up varied with the clinical scenarios described (Table 1). For patients with trunk or extremity tumours at low risk of relapse, answers ranged from 1 year to lifelong with 5 years the most common response (44%) and 10 years the second most common (23%). For those with trunk or extremity tumours at high-risk answers ranged from 5 years to lifelong with 10 years the most common response (36%) and 5 years less common (22%). Responses for patients with retroperitoneal or abdominal tumours were similar to the high-risk responses, with answers ranging from 4 years to lifelong, 10 years the most common response (38%) and 5 years second most common (19%).

Clinic visits and chest X-rays were the most common methods for follow up. The frequency of clinic visits declined with time (Table 2). The most common pattern for patients with low-risk trunk or extremity tumours in the first 5 years was 4 visits in years 1 and 2 and 2 visits in years 3, 4, and 5 (14%; 12/88). This pattern was also the most frequent for patients with high-risk trunk or extremity tumours (18%; 19/99) and retroperitoneal/abdominal tumours (13%; 8/63). The median total number of clinic visits in 5 years was 12 (5 to 30) for low-risk trunk or extremity tumours, 14 (6 to 27) for high risk trunk and extremity tumours and 14 (5 to 30) for retroperitoneal or abdominal tumours.

The use of radiological investigations and routine blood tests for follow up is summarised in Table 3. All investigations were used more often for high-risk compared with low-risk trunk or extremity tumours. There was wide variation in the use of these investigations. Most respondents (>85%) specified the use of at least one chest x-ray during follow up but responses ranged from no chest x-rays requested to as many as 24 in the first 5 years of follow up. Chest CT scans were used less frequently for follow up, being used in 16% of low-risk trunk or extremity tumours, 29% of high-risk trunk or extremity tumours and 25% of abdominal or retroperitoneal tumours. Local imaging investigations were used for follow up for just 38% of patients with low-risk tumours but 61% of patients with high-risk and 73% of patients with abdominal or retroperitoneal tumours. Few respondents (<10%) routinely used blood tests in follow up.

### 3.5. Attitudes to follow up

Of the 121 survey respondents, 106 completed questions on attitudes to follow up with the remaining 15 not responding, possibly due to lack of involvement either in follow up or in adult patients. Despite the variation in practice, 88/106 (83%) of respondents thought that regular follow up was of benefit for patients with soft tissue sarcoma, with only 3 stating that they did not think it was of benefit and the remaining 15 not sure. Forty (38%) believed that detecting metastases before they become symptomatic leads to improved survival for patients, with 45 (42%) not sure. Seventy-one (67%) thought that detecting a local recurrence before it became symptomatic was of benefit to patients, with 26 (25%) not sure.

This final part of this section also asked the respondents their opinion about carrying out a randomised trial of follow-up protocols in adult patients who have been treated for soft tissue sarcoma possibly with randomisation according to the risk of relapse. Of 106 survey respondents, 96 (91%) felt a trial of this kind to be worth taking forward with 85 (80%) prepared to enter patients into such a trial. In terms of follow-up protocols, at the one extreme 53/106 (50%) would not have a problem with an intensive follow-up regime involving regular chest CT and/or local site imaging and at the other extreme 57/106 (54%) thought it would be reasonable to follow up selected patients in the community.

### 3.6. Estimated variation in costs

The mean costs of the stated practice for follow up for low-risk trunk or extremity tumours was £2,542 (£372 to £7,852; n=64), for high-risk trunk or extremity tumours was £3,548 (£1,091 to £7,961; n=83), and for retroperitoneal and abdominal tumours was £3,876 (£595 to £7,961; n=45).

## 4. DISCUSSION

This paper has identified for the first time how patients with soft tissue sarcoma are followed up after treatment in the United Kingdom. We undertook a questionnaire survey of hospital clinicians with a declared interest in the management of sarcomas. In the past decade, there has been a move to manage all malignancies through regionally accredited multidisciplinary teams (MDT). Sarcoma services have lagged behind the more common solid tumours in this process, so patients with soft tissue sarcomas are often treated by nonspecialist orthopaedic, plastic, or general surgeons (Clasby et al. [13]; Glencross et al. [14]). Our survey included only those with a declared interest in sarcoma, of whom 91% had access to an MDT. This, therefore, represents best practice in the UK, and likely excludes those units where sarcomas are treated by generalists. Any questionnaire survey has the potential weakness of nonresponse bias but the response rate in our study was high and the level of completeness was good. Therefore, we believe the results are representative of UK clinicians involved in follow up for sarcoma.

We have shown that most clinicians follow up their patients, most frequently with clinic visits and chest x-rays. Chest CT scanning and local site imaging, such as MRI or CT, are used less frequently; and blood tests seldom used. As expected, the frequency of clinic visits and investigations declines with time but we have shown considerable variation in practice, and therefore in the cost of follow up. Our study was not designed to look at the differences in follow-up practices between different medical specialties.

Follow up after treatment of a malignant tumour serves a number of purposes. The major goal is the detection of local or systemic recurrence of disease, but other goals include the reassurance of patients, the collection of data about outcomes, management of the late effects of treatment, and the detection of second malignancies or other unrelated medical conditions (Brennan [15]). Although follow-up protocols may be driven by the concept that early physician-lead detection of recurrence leads to improved survival, there is little evidence for this (Brennan [15]). Our respondents reflected this uncertainty. Although the majority felt that follow up was of benefit after treatment of soft tissue sarcoma, there was less certainty about whether or not early detection of local recurrence or asymptomatic chest metastases benefits patients.

The clinician's choice of follow-up protocol is likely to be influenced by factors such as training, age, the availability of clinic time, and whether or not there are appropriate treatments for relapsed patients (Sakata et al. [10]). Our study confirms that clinicians in the United Kingdom also vary follow up according to the perceived risk of relapse, which is similar to the results of the survey by the American Society of Surgical Oncology (Sakata et al. [11]). Respondents were asked to state which factors they associated with disease relapse. In our survey, the three factors most often selected by respondents as associated with local recurrence were surgical margin status, histological grade, and tumour size. Published series support surgical margin status and histological grade as predictors of local recurrence; there is less support for tumour size as a predictor (Coindre et al. [16]; Pisters et al. [17]; Stojadinovic et al. [18]; Trovik et al. [19]).

Respondents selected histological grade, tumour size, and histological type most often as risk factors for metastatic disease. The literature supports the view that the main risk factors for metastatic disease and overall survival are tumour grade, size, and depth with some influence also of tumour diagnosis, site, and patient age. This has been incorporated into a nomogram, which is also available online (Kattan et al. [5]).

Follow up is expensive; variation in protocols results in significant variation in costs. Although our cost model is somewhat simplistic and does not account for the cost of treatment of relapse or allow for drop out because of death, it shows a seven-to-twenty fold difference in follow-up costs between the most and the least intensive regimens depending on the type of clinical scenario. This is less extreme than the experience in the United States where a 42.8-fold variation in cost was identified ($485–$21,235; mean cost $6,401) (Goel et al. [8]) but still is indicative of the large effect on cost that results from wide variability in practice. It is likely that many of these follow-up regimes are not cost effective.

Patients are good at detecting local recurrences of tumours in the trunk and extremity; most local recurrences are detected either by the patient or their primary care physician between clinic visits (Kattan et al. [5]; Whooley et al. [20]). Local site imaging is a low-yield investigation, detecting only one of 29 local recurrences in a series in which selected patients with high-grade tumours received local site imaging annually (Whooley et al. [20]). The impact of regular local site imaging for all patients is not known, but might be expected to lead to earlier detection of local recurrence in some patients at the expense of a number of false-positive scans in others.

Following detection of locally recurrent soft tissue sarcoma in the extremities or trunk, adequate local therapy can lead to prolonged survival (Moureau-Zabotto et al. [21]; Trovik [6]), although these patients are at increased risk of systemic relapse (Ramanathan et al. [22]). Complete resection of the local recurrence is an important part of this treatment, but whether regular surveillance increases the likelihood of success in this regard is not clear. Treatment of a local recurrence is more likely to require amputation compared with treatment of a primary tumour (Trovik [6]) and it is possible that early detection of local recurrence might prevent this in a number of cases or could lead to improved function of the salvaged extremity. Adjuvant radiotherapy is recommended following subablative resection of locally recurrent disease, but this too can result in long-term impairment of function.

Local recurrences of tumours in the retroperitoneum or abdomen are more difficult to detect clinically and our survey confirms that local imaging is more widely used in this setting. Salvaging patients with locally recurrent tumours in these anatomical sites is difficult, although some patients with low-grade tumours can have repeated debulking surgery over several years. Once more, the value of earlier detection in this situation has not been determined.

Screening for asymptomatic lung metastases is controversial. In our survey, chest X-rays were used more frequently than chest CT scans for the detection of metastases. The former are cheaper and can be done in the clinic. The use of chest CT scans might be expected to lead to the earlier detection of metastases, but is associated with greater radiation exposure to patients. There is evidence that a significant proportion of patients with metastases can achieve long-term survival with appropriate treatment (van Geel et al. [23]). In one study, 248 of 719 patients presenting with pulmonary metastases underwent surgical resection (Weiser et al. [24]). These patients remain at risk of further relapse, but even second and third relapses can, on occasion be treated successfully (Weiser et al. [24]). Much seems to depend on whether or not the metastases can be completely removed at the time of surgery; patients with unresectable metastases are incurable (Billingsley et al. [25]; Casson et al. [26]; van Geel et al. [23]). Given the lack of randomised studies, there are no data to indicate a survival advantage for metastectomy. Patients most likely to be salvaged after developing lung metastases are younger, have a grade one or two primary tumour, and present with a solitary metastases after a long disease-free interval and which can be widely resected (Billingsley et al. [25]; van Geel et al. [23]). Cytotoxic chemotherapy is effective for some (objective response rates less than 20%) but the median survival is only 12 months (van Glabbeke et al. [27]).

The variation in practice and cost shown in this survey suggest a role for a randomised study to determine the optimum strategy for follow up of this group of patients. Such a study should consider the economic cost of follow up, the psychological impact on the patient, and whether more intensive regimes lead to earlier detection of local or systemic disease than less intensive regimes. Such a study might also investigate the most effective imaging modalities for detection of local or systemic disease and the impact of patient education programs. The impact of earlier detection and, therefore, treatment on overall survival and on the function or preservation of an extremity after treatment for local recurrence should also be considered. Resolving these questions into a single study may be challenging.

## 5. CONCLUSION

This study has defined for the first time how patients who have been treated for soft tissue sarcoma in the United Kingdom are followed up after treatment. We have demonstrated that there is wide variation in practice and cost. Follow up is clearly a question of balancing a number of objectives, including maximising survival, quality of life, psychological outcomes, and function. Given that there is little evidence for one follow up protocol over another, there is clearly potential for developing a study of these issues in the United Kingdom.

## Figures and Tables

**Table 1:** Length of follow up after treatment.

	Low risk trunk or extremity tumours (N=88)	High risk trunk or extremity tumours (N=99)	Abdominal or retroperitoneal tumours (N=63)
Less than 5 years	3 (3%)	0 (0%)	1 (2%)
Exactly 5 years	39 (44%)	22 (22%)	12 (19%)
At least 5 years	7 (8%)	9 (9%)	6 (10%)
Exactly 8 years	1 (1%)	2 (2%)	2 (3%)
Exactly 10 years	20 (23%)	36 (36%)	24 (38%)
At least 10 years	6 (7%)	6 (6%)	4 (6%)
At least 15 years	0 (0%)	1 (1%)	0 (0%)
Until adulthood	1 (1%)	2 (2%)	1 (2%)
Lifelong	9 (10%)	15 (15%)	10 (16%)
No response	2 (2%)	6 (6%)	3 (5%)

**Table 2:** Number of clinic visits per year after treatment by clinical scenario.

	Median (range) number of clinic visits per year after treatment					
Clinical scenario	Year 1	Year 2	Year 3	Year 4	Year 5	Each year thereafter
Low-risk trunk or extremity tumours	4 (1–12)	3 (1–6)	2 (1–6)	2 (1–6)	1 (1–6)	1 (0–2)
High-risk trunk or extremity tumours	4 (2–12)	4 (1–6)	2 (1–4)	2 (1–6)	2 (1–6)	1 (0–2)
Abdominal or retroperitoneal tumours	4 (2–12)	4 (1–6)	2 (1–6)	2 (1–6)	2 (0–6)	1 (0–2)

**Table 3:** The use of radiological investigations or blood tests for follow up.

	Chest x-rays		Chest CT scans		Local imaging	
Clinical scenario	At least one	Median number in 5 years (range)	At least one	Median number in 5 years (range)*	At least one	Median number in 5 years (range)*	Routine blood tests
Low-risk trunk or extremity tumours	76/88 (86%)	8 (0–24)	14/88 (16%)	0 (0–6)	33/88 (38%)	1 (0–13)	5/88 (6%)
	(5 no response)		(15 no response)		(13 no response)		

High risk trunk or extremity tumours	90/99 (91%)	13 (0–24)	29/99 (29%)	0 (0–10)	60/99 (61%)	2 (0–13)	8/99 (8%)
	(8 no response)		(15 no response)		(12 no response)		

Abdominal or	55/63 (87%)	12 (0–24)	16/63 (25%)	0 (0–9)	46/63 (73%)	5 (0–13)	6/63 (10%)
retroperitoneal tumours	(6 no response)		(10 no response)		(7 no response)		

*Some respondents specified that CT scans and local imaging should only be performed when clinically indicated rather than routinely.
